# Transcriptional coactivator PGC-1α contributes to decidualization by forming a histone-modifying complex with C/EBPβ and p300

**DOI:** 10.1016/j.jbc.2022.101874

**Published:** 2022-03-28

**Authors:** Haruka Takagi, Isao Tamura, Taishi Fujimura, Yumiko Doi-Tanaka, Yuichiro Shirafuta, Yumiko Mihara, Ryo Maekawa, Toshiaki Taketani, Shun Sato, Hiroshi Tamura, Norihiro Sugino

**Affiliations:** Department of Obstetrics and Gynecology, Yamaguchi University Graduate School of Medicine, Ube, Japan

**Keywords:** peroxisome proliferator–activated receptor gamma coactivator 1-alpha (PGC-1α), CCAAT/enhancer-binding protein beta (C/EBPβ), histone acetylation, endometrial stromal cell, decidualization, genome editing, epigenetics, E1A-binding protein p300 (p300), C/EBPβ, CCAAT/enhancer-binding protein beta, Cas9, CRISPR-associated protein 9, ChIP, chromatin immunoprecipitation, co-IP, coimmunoprecipitation, DMEM, Dulbecco's modified Eagle's medium, ESC, endometrial stromal cell, FBS, fetal bovine serum, H3K27ac, acetylation of histone-H3 lysine-27, HAT, histone acetyltransferase, IGFBP-1, insulin-like growth factor–binding protein-1, PGC-1α, peroxisome proliferator-activated receptor gamma coactivator 1 alpha, PRL, prolactin, sgRNA, single guide RNA, TSS, transcription start site

## Abstract

We previously reported that CCAAT/enhancer-binding protein beta (C/EBPβ) is the pioneer factor inducing transcription enhancer mark H3K27 acetylation (H3K27ac) in the promoter and enhancer regions of genes encoding insulin-like growth factor–binding protein-1 (IGFBP-1) and prolactin (PRL) and that this contributes to decidualization of human endometrial stromal cells (ESCs). Peroxisome proliferator–activated receptor gamma coactivator 1-alpha (PGC-1α; *PPARGC1A*) is a transcriptional coactivator known to regulate H3K27ac. However, although PGC-1α is expressed in ESCs, the potential role of PGC-1α in mediating decidualization is unclear. Here, we investigated the involvement of PGC-1α in the regulation of decidualization. We incubated ESCs with cAMP to induce decidualization and knocked down PPARGC1A to inhibit cAMP-induced expression of IGFBP-1 and PRL. We found cAMP increased the recruitment of PGC-1α and p300 to C/EBPβ-binding sites in the promoter and enhancer regions of IGFBP-1 and PRL, corresponding with increases in H3K27ac. Moreover, PGC-1α knockdown inhibited these increases, suggesting PGC-1α forms a histone-modifying complex with C/EBPβ and p300 at these regions. To further investigate the regulation of PGC-1α, we focused on C/EBPβ upstream of PGC-1α. We found cAMP increased C/EBPβ recruitment to the novel enhancer regions of PPARGC1A. Deletion of these enhancers decreased PGC-1α expression, indicating that C/EBPβ upregulates PGC-1α expression by binding to novel enhancer regions. In conclusion, PGC-1α is upregulated by C/EBPβ recruitment to novel enhancers and contributes to decidualization by forming a histone-modifying complex with C/EBPβ and p300, thereby inducing epigenomic changes in the promoters and enhancers of IGFBP-1 and PRL.

Human endometrial stromal cells (ESCs) undergo cyclic changes during the menstrual cycle in response to changing levels of steroid hormones. Decidualization is one of these changes in which ESCs differentiate into decidual cells by the action of progesterone, which is essential for implantation and maintenance of pregnancy (1, 2, 3). During decidualization, a number of genes are upregulated by the activation of various transcription factors (4, 5, 6). Our previous genome-wide analyses revealed that a number of genes are upregulated or downregulated by decidualization in human ESCs (4, 7). Gene expression including transcription involves a change of chromatin structure, which can be regulated by epigenetic mechanisms such as histone modifications (8, 9, 10). Acetylation of histone-H3 lysine-27 (H3K27ac) is one of the histone modifications that activate gene transcription and is highly enriched in the active promoter or enhancer regions (11). We also found that H3K27ac levels increase throughout the genome during decidualization and that upregulation of the expressions of many genes was accompanied by increases of H3K27ac levels (4, 7, 12).

In general, chromatin remodeling regulated by histone modifications is induced by the recruitment of pioneer transcription factors, which is responsible for the initiation of the epigenetic changes (13, 14). We reported that CCAAT/enhancer-binding protein beta (C/EBPβ) works as the pioneer factor at the promoter and enhancer regions of insulin-like growth factor–binding protein-1 (IGFBP-1) and the promoter region of prolactin (PRL), which are specific markers of decidualization (15, 16, 17). Furthermore, we recently reported that C/EBPβ was involved in the genome-wide increase of H3K27ac and the upregulation of a number of genes during decidualization (4). These facts indicated that genome-wide epigenomic changes induced by C/EBPβ are essential for decidualization.

When pioneer factors induce histone acetylation and chromatin remodeling, they must form a complex at the genomic locus with cofactors that have histone acetyltransferase (HAT) activities (18, 19). This complex is called the histone-modifying complex. p300 is one of the HATs that form a histone-modifying complex with C/EBPβ (20). We have previously reported that p300 is a HAT that induces H3K27ac by cooperating with C/EBPβ at the IGFBP-1 enhancer region (21). However, a histone-modifying complex contains several cofactors other than pioneer factors and HATs. These cofactors are also essential to form a histone-modifying complex and activate transcription (22, 23, 24). Therefore, it is important to identify cofactors of C/EBPβ that are involved in the induction of H3K27ac during decidualization.

Peroxisome proliferator–activated receptor gamma coactivator 1 alpha (PGC-1α) is a transcription cofactor that regulates various physiological functions, such as mitochondrial biogenesis, thermogenesis, metabolism, and fatty acid oxidation in several organs, including reproductive organs (25, 26, 27, 28, 29, 30, 31). PGC-1α is also expressed in human ESCs, and its expression increases with decidualization (32). However, the physiological role of PGC-1α in the endometrium is unclear. Although PGC-1α itself does not have HAT activity, it recruits cofactors with HAT activities, such as p300 and steroid receptor coactivator-1, and contributes to the induction of histone acetylation (33, 34, 35, 36). It should be noted that PGC-1α has no DNA-binding domain so that it must interact with other transcription factors such as C/EBPβ to bind to promoter and enhancer regions (37, 38, 39). These facts led us to hypothesize that PGC-1α works as a cofactor of C/EBPβ to induce histone acetylation during decidualization.

In this study, we showed that PGC-1α forms a histone-modifying complex with C/EBPβ and p300 and induces H3K27ac at the promoter and enhancer regions of IGFBP-1 and the promoter region of PRL in ESCs during decidualization. In addition, we show how PGC-1α is upregulated by decidualization.

## Results

### PGC-1α expression in human endometrium and ESCs and the effect of cAMP on PGC-1α expression

**Figure 1:**
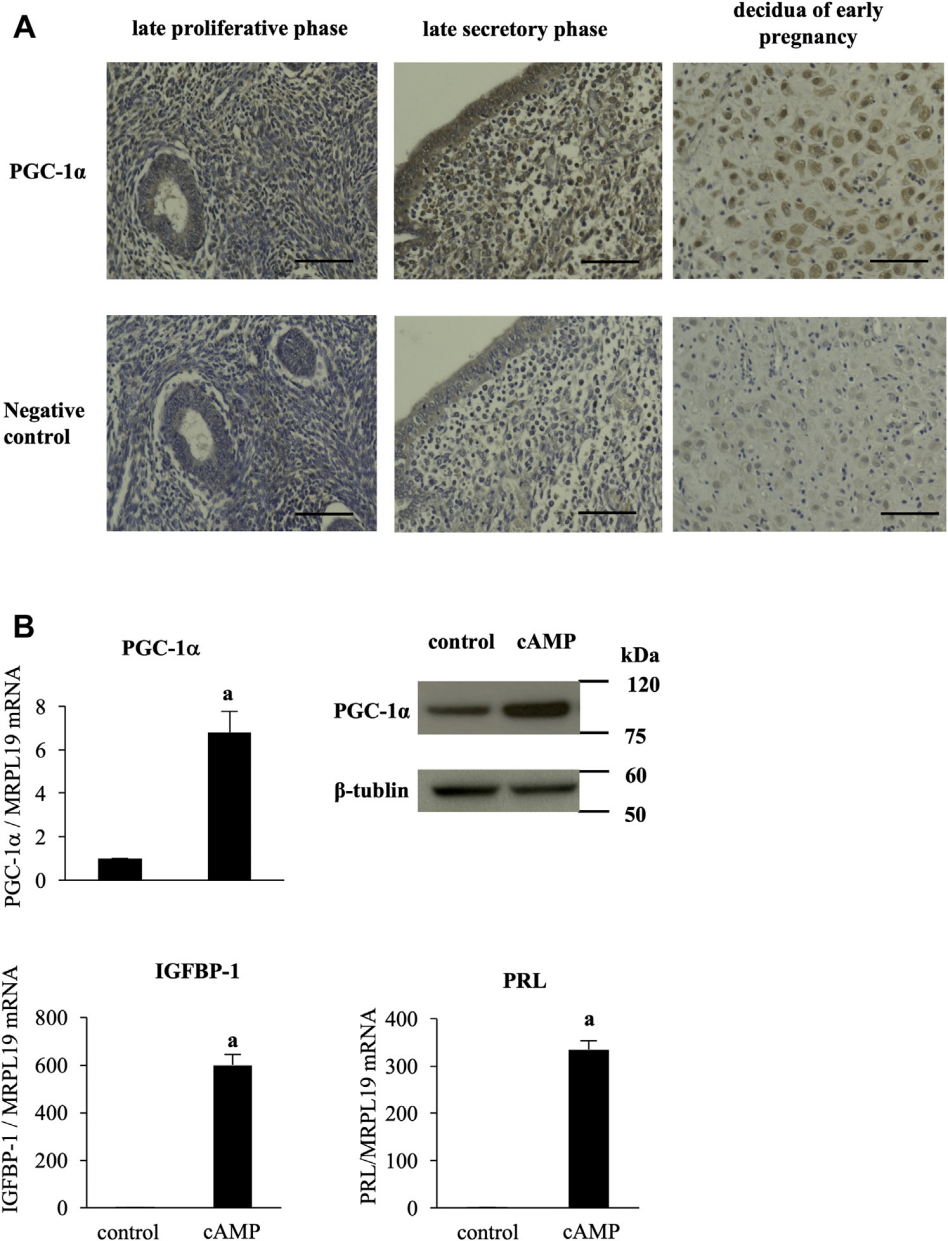
**PGC-1α expression in human endometrium and ESCs and the effect of cAMP on PGC-1α expression.***A*, immunohistochemical expression of PGC-1α in the late proliferative and secretory phase endometrium and decidua of early pregnancy. The photographs in the lower row are negative controls in each sample. Scale bars, 50 μm. *B*, ESCs were treated with or without cAMP (0.5 mM) for 4 days. Cells treated without cAMP were used as the control. mRNA expression was analyzed by quantitative real-time RT-PCR. Values of PGC-1α, IGFBP-1, and PRL were normalized to those of MRPL19 and expressed as a ratio of the control sample. SDs of control samples are shown in Table S4. Values are mean ±SD of three different incubations. *A*, *p* < 0.01 *versus* control. Whole-cell lysates were prepared and subjected to Western blotting to examine PGC-1α protein expression. β-Tubulin was used as an internal control. The immunoblot is a representative of three different incubations. ESC, endometrial stromal cell; IGFBP-1, insulin-like growth factor–binding protein-1; PGC-1α, peroxisome proliferator-activated receptor gamma coactivator 1 alpha; PRL, prolactin.

The expression of PGC-1α in the human endometrium was examined by immunohistochemistry. PGC-1α was expressed in stromal cells obtained from the proliferative phase endometrium (Fig. 1*A*). Stronger expression of PGC-1α was observed in the stromal cells that were morphologically identified as predecidual cells in the late secretory phase endometrium and decidual cells of early pregnancy. The expression of PGC-1α in the human ESCs was also examined by real-time RT-PCR and Western blotting. cAMP increased the mRNA and protein expression levels of PGC-1α with the induction of IGFBP-1 and PRL mRNA in ESCs (Fig. 1*B*).

### Involvement of PGC-1α in cAMP-induced gene expression of IGFBP-1 and PRL

**Figure 2:**
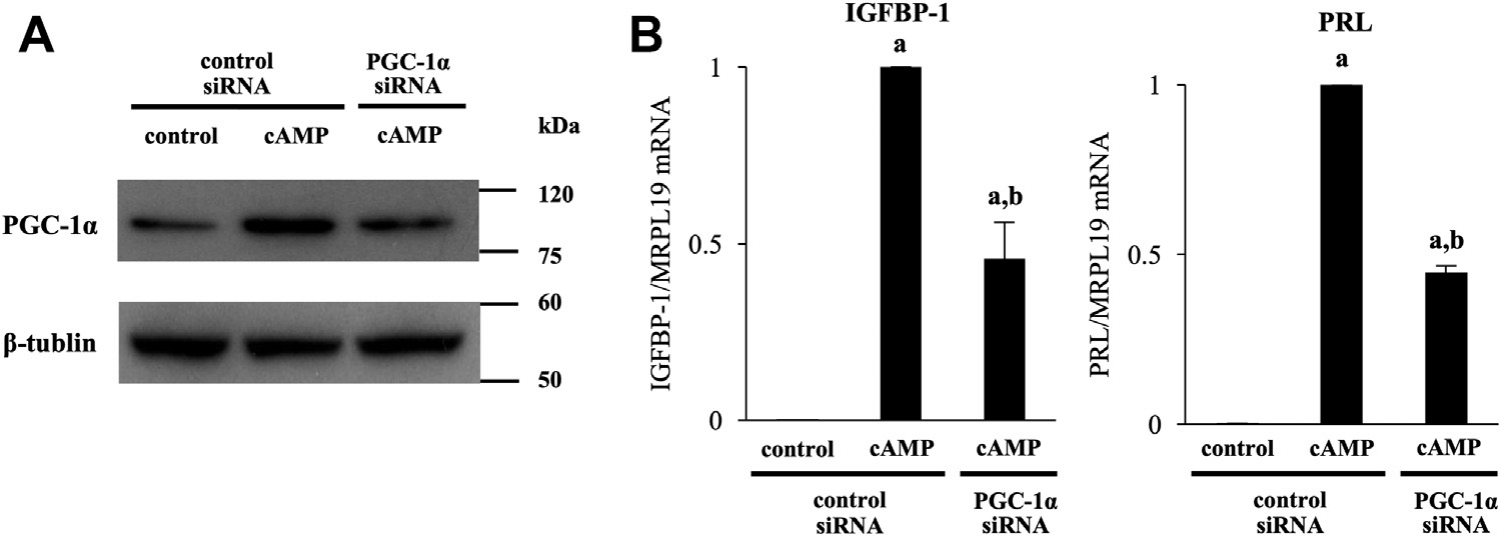
**Involvement of PGC-1α in cAMP-induced gene expression of IGFBP-1 and PRL.***A*, ESCs were transfected with a siRNA targeted against PGC-1α or with a nontargeting siRNA as a control. Forty-eight hours after siRNA transfection, ESCs were treated with or without cAMP for 4 days. Whole-cell lysates were prepared and subjected to Western blotting to examine the knockdown of PGC-1α protein expression. β-Tubulin was used as an internal control. The immunoblot is a representative of three different incubations. *B*, mRNA expression was analyzed by quantitative real-time RT-PCR. Values of IGFBP-1 and PRL were normalized to those of MRPL19 and expressed as a ratio of the control sample. SDs of control samples are shown in Table S4. Values are mean ±SD of three different incubations. *A*, *p* < 0.01 *versus* control treatment in the control siRNA; *B*, *p* < 0.01 *versus* cAMP treatment in the control siRNA. ESC, endometrial stromal cell; IGFBP-1, insulin-like growth factor–binding protein-1; PGC-1α, peroxisome proliferator–activated receptor gamma coactivator 1 alpha; PRL, prolactin.

To examine whether the stimulatory effects of cAMP on mRNA expression of IGFBP-1 and PRL are mediated through PGC-1α, PGC-1α was knocked down by small interfering RNA (siRNA). The increase in PGC-1α protein expression by cAMP was suppressed by siRNA treatment (Fig. 2*A*). Knockdown of PGC-1α significantly inhibited cAMP-induced expressions of IGFBP-1 and PRL (Fig. 2*B*). The morphological changes induced by decidualization stimulus with cAMP were not altered by PGC-1α knockdown (data not shown).

### cAMP-induced recruitments of PGC-1α and p300 to the promoter and enhancer regions of IGFBP-1 and the promoter region of PRL

**Figure 3:**
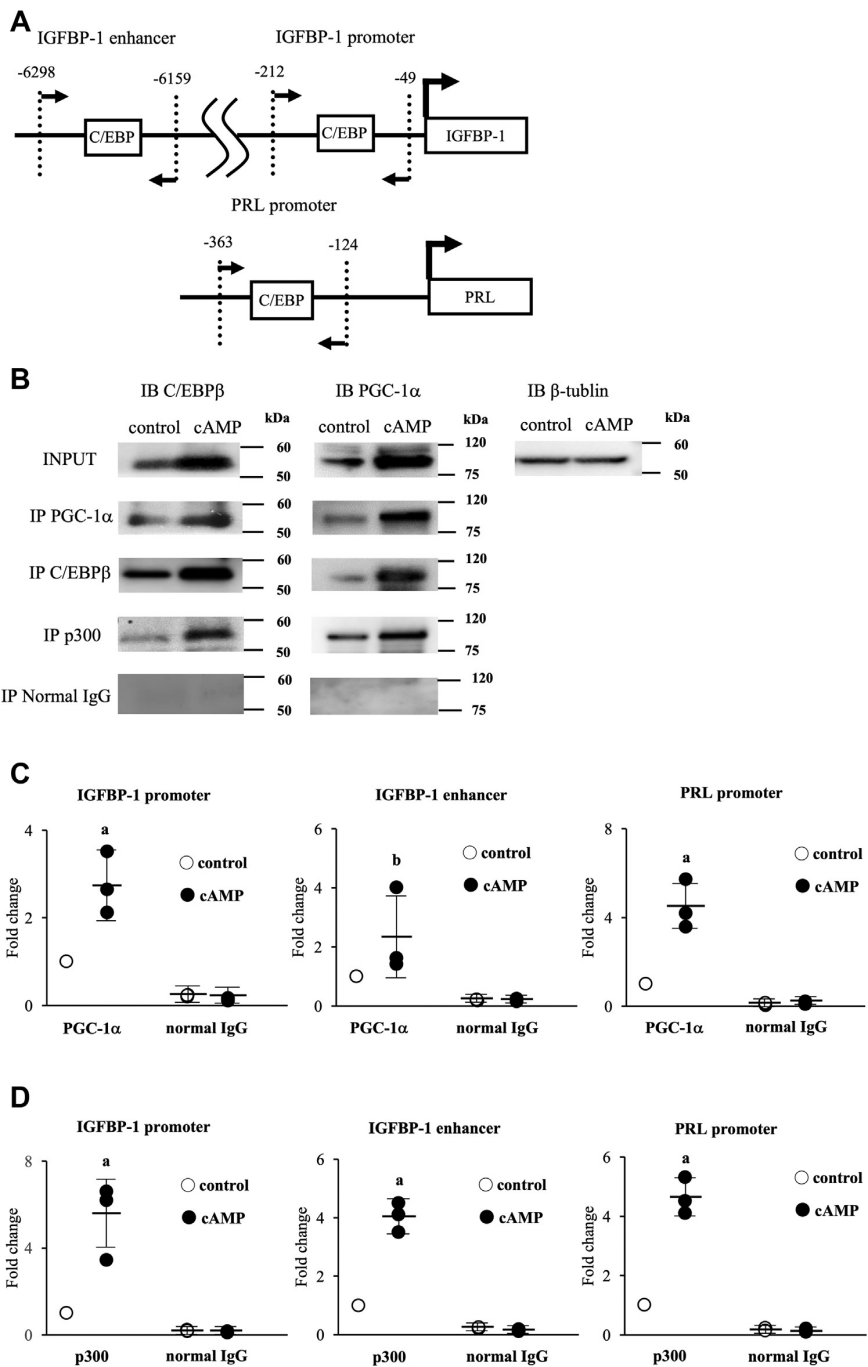
**cAMP-induced PGC-1α and p300 recruitment to the promoter and enhancer regions of IGFBP-1 and the promoter region of PRL.***A*, locations of the C/EBPβ-binding sites in the promoter and enhancer regions of IGFBP-1 and the promoter region of PRL. For the ChIP assay, three regions were amplified by PCR: -49 to -212 bp for the IGFBP-1 promoter, -6159 to -6298 bp for the IGFBP-1 enhancer, -124 to -363 bp for the PRL promoter. *B*, co-immunoprecipitation of PGC-1α, C/EBPβ, and p300. ESCs were treated with or without cAMP for 4 days. Whole-cell lysates were prepared and subjected to immunoprecipitation (IP) with a specific PGC-1α, C/EBPβ, and p300 antibody. Normal rabbit IgG was used as a negative control for IP. Immunoprecipitated proteins were separated on SDS-polyacrylamide gels together with samples from the input (10% amount used for IP). Immunoblot (IB) was done with anti-PGC-1α or C/EBPβ antibody. β-Tubulin was used as an internal control. *C* and *D*, recruitment levels of PGC-1α and p300 to the promoter and enhancer regions of IGFBP-1 and the promoter region of PRL were analyzed by ChIP assay. Primers were designed surrounding the C/EBPβ-binding sites. Normal rabbit IgG was used as a negative control. The relative recruitment levels were analyzed by real-time PCR. Values were expressed as a ratio of control sample. SDs of control samples are shown in Table S4. Data are mean ±SD of three different incubations. *A*, *p* < 0.01 *versus* control. *B*, *p* < 0.01 *versus* control. C/EBPβ, CCAAT/enhancer-binding protein beta; ESC, endometrial stromal cell; IGFBP-1, insulin-like growth factor–binding protein-1; PGC-1α, peroxisome proliferator–activated receptor gamma coactivator 1 alpha; PRL, prolactin.

The promoter regions of IGFBP-1 and PRL are important for cAMP-induced expressions because there are many *cis*-regulatory elements, including C/EBPβ-binding regions (Fig. 3*A*) (10, 17, 40, 41, 42, 43, 44). We recently identified an enhancer region of IGFBP-1 approximately 5000 bp upstream from the transcription start site (TSS) (Fig. 3*A*) (21). C/EBPβ binds to these promoter and enhancer regions to upregulate their expressions during decidualization (10, 17, 21, 45). Although PGC-1α does not have DNA-binding domains, it can access the promoter and enhancer regions by interacting with C/EBPβ (38, 39). Therefore, we hypothesized that PGC-1α is recruited to C/EBPβ-biding sites in these promoter and enhancer regions through the interaction with C/EBPβ. First, by performing a coimmunoprecipitation (co-IP) assay, we confirmed that PGC-1α interacts with C/EBPβ both in nondecidualized ESCs and decidualized ESCs (Fig. 3*B*). To examine the PGC-1α recruitments to the promoter and enhancer regions of IGFBP-1 and the promoter region of PRL, chromatin immunoprecipitation (ChIP) primers were designed to cover the C/EBPβ-binding sites that we previously identified (Fig. 3*A*) (10, 17, 21, 40). cAMP significantly increased the PGC-1α recruitments to these C/EBPβ-binding sites (Fig. 3*C*). We previously reported that cAMP increases the H3K27ac levels of the promoter and enhancer regions of IGFBP-1 and the promoter region of PRL (17, 21). p300 is a transcription coactivator that has HAT activities and induces H3K27ac (46, 47). p300 is also reported to interact with both PGC-1α and C/EBPβ (48, 49, 50). We confirmed that p300 interacts with both PGC-1α and C/EBPβ in nondecidualized ESCs and decidualized ESCs (Fig. 3*B*). Therefore, we examined the effect of cAMP on the p300 recruitment. cAMP significantly increased the p300 recruitment to these regions (Fig. 3*D*).

### Involvement of PGC-1α in the changes of H3K27ac status of the promoter and enhancer regions of IGFBP-1 and the promoter region of PRL

**Figure 4:**
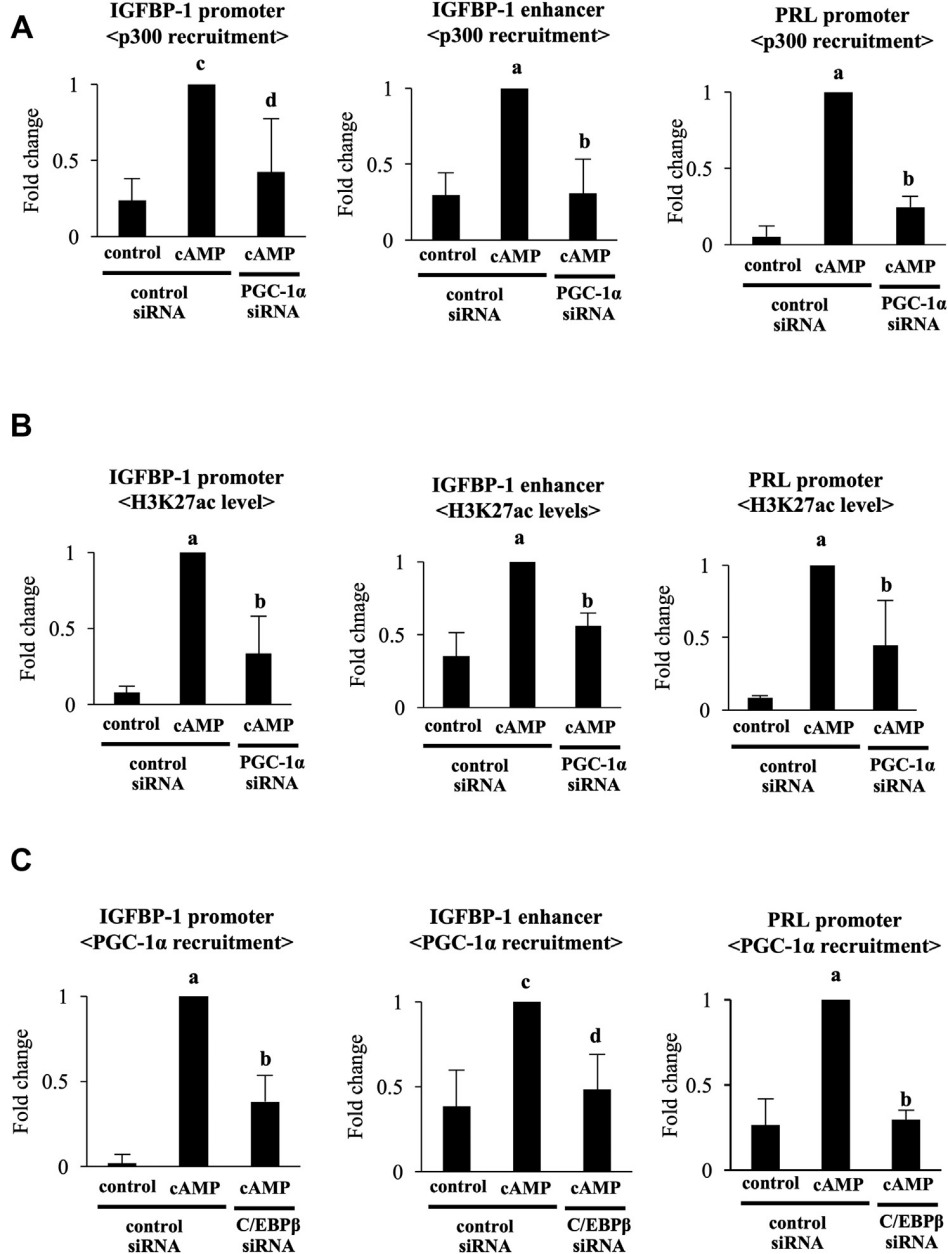

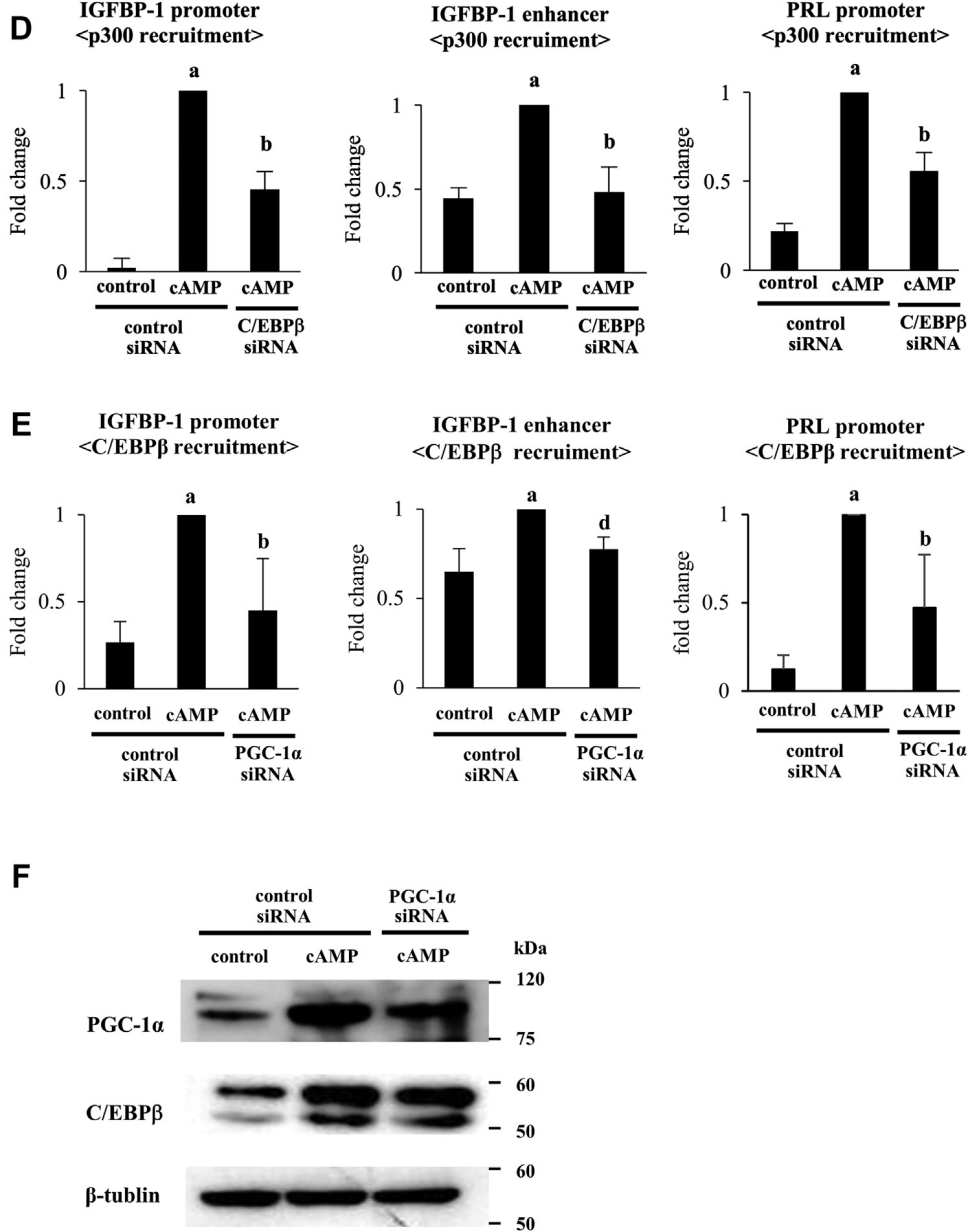
**Involvement of PGC-1α in the increase of H3K27ac levels in the promoter and enhancer regions of IGFBP-1 and the promoter region PRL.** ESCs were transfected with a siRNA targeted against PGC-1α, C/EBPβ, or with a nontargeting siRNA as a control. Forty-eight days after siRNA transfection, ESCs were treated with or without cAMP for 4 days. The levels of p300 recruitment (*A* and *D*), H3K27ac (*B*), PGC-1α recruitment (*C*), and C/EBPβ recruitment (*E*) in the promoter and enhancer regions of IGFBP-1 and the promoter regions of PRL were analyzed by ChIP assay. The relative recruitment levels were analyzed by real-time PCR. Values were expressed as a ratio of cAMP treatment in the control siRNA sample. SDs of cAMP treatment in the control siRNA sample are shown in Table S4. Data are mean ±SD of three different incubations. *A*, *p* < 0.01 *versus* control treatment in the control siRNA; *B*, *p* < 0.01 *versus* cAMP treatment in the control siRNA. *C*, *p* < 0.05 *versus* control treatment in the control siRNA; *D*, *p* < 0.05 *versus* cAMP treatment in the control siRNA. *F*, whole-cell lysates were prepared and subjected to Western blotting to examine the effect of PGC-1α knockdown on C/EBPβ protein expression. β-Tubulin was used as an internal control. The immunoblot is a representative of three different incubations. C/EBPβ, CCAAT/enhancer-binding protein beta; ChIP, chromatin immunoprecipitation; ESC, endometrial stromal cell; H3K27ac, acetylation of histone-H3 lysine-27; IGFBP-1, insulin-like growth factor–binding protein-1; PGC-1α, peroxisome proliferator–activated receptor gamma coactivator 1 alpha; PRL, prolactin.

Because PGC-1α and p300 colocalized on the C/EBPβ-binding sites in the promoter and enhancer regions of IGFBP-1 and the promoter region of PRL, we hypothesized that PGC-1α is involved in the p300 recruitment and H3K27ac levels by interacting with C/EBPβ in these regions. To test this hypothesis, effects of PGC-1α knockdown on the levels of p300 recruitment and H3K27ac were examined. The increases of p300 recruitments and H3K27ac levels induced by cAMP were significantly inhibited by PGC-1α knockdown (Fig. 4, *A* and *B*). In addition, C/EBPβ knockdown significantly inhibited cAMP-increased recruitments of PGC-1α and p300 (Fig. 4, *C* and *D*). We also examined whether PGC-1α regulates C/EBPβ recruitments to these regions. cAMP significantly increased the C/EBPβ recruitments to these regions, which was inhibited by PGC-1α knockdown (Fig. 4*E*). Knockdown of PGC-1α did not affect the cAMP-increased C/EBPβ protein expression (Fig. 4*F*).

### Involvement of C/EBPβ in the cAMP-increased gene expression of PGC-1α

**Figure 5:**
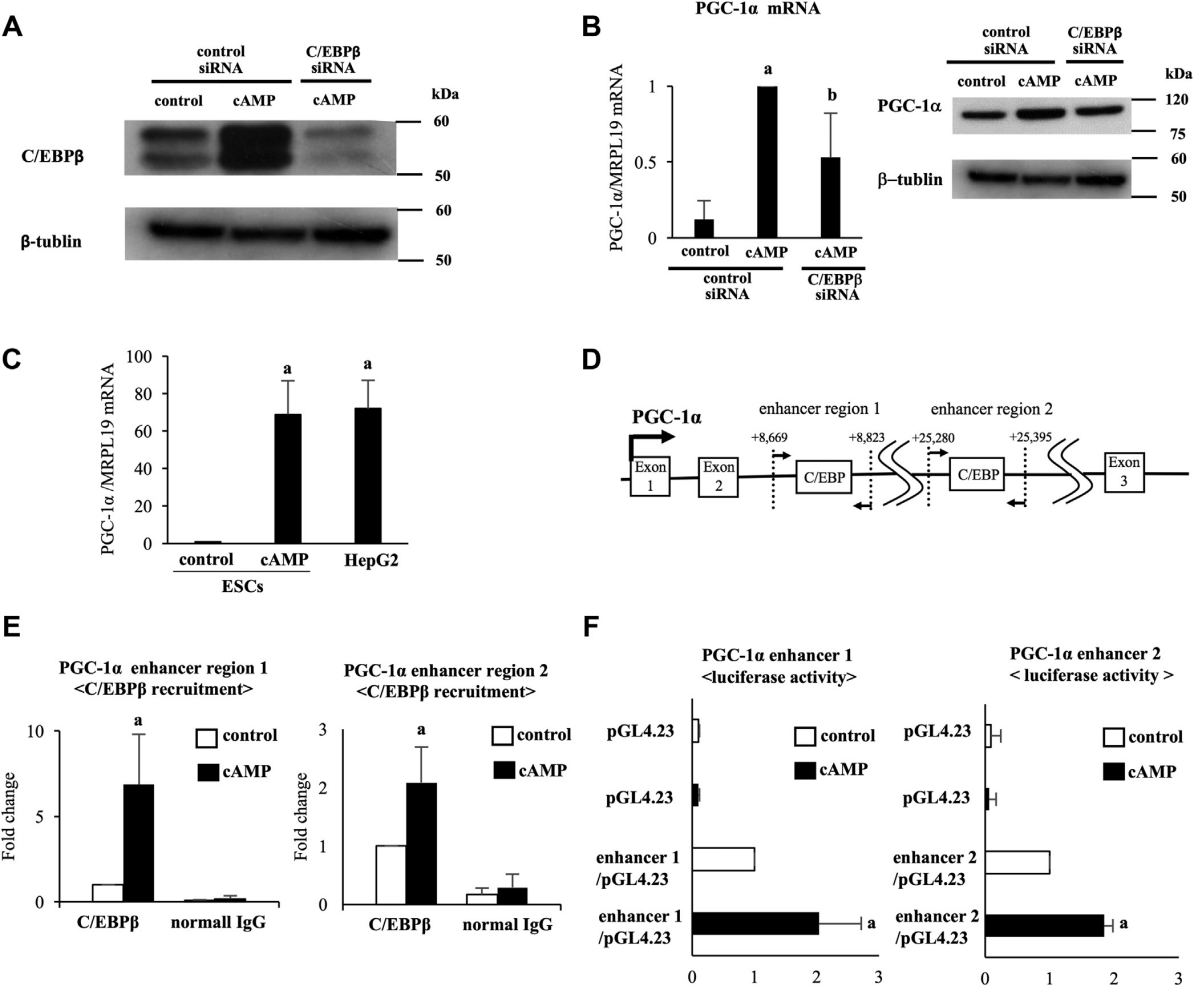
**Involvement of C/EBPβ in the cAMP-increased gene expression of PGC-1α**. *A*, ESCs were transfected with a siRNA targeted against C/EBPβ or with a nontargeting siRNA as a control. Forty-eight days after siRNA transfection, ESCs were treated with or without cAMP for 4 days. Whole-cell lysates were prepared and subjected to Western blotting to examine the knockdown of C/EBPβ protein expression. β-Tubulin was used as an internal control. The immunoblot is a representative of three different incubations. *B*, PGC-1α mRNA expression was analyzed by quantitative real-time RT-PCR. Values of PGC-1α were normalized to those of MRPL19 and expressed as a ratio of the cAMP treatment sample in the control siRNA. SD of cAMP treatment in the control siRNA sample is shown in Table S4. Values are mean ±SD of three different incubations. *A*, *p* < 0.01 *versus* control treatment in the control siRNA; *B*, *p* < 0.05 *versus* cAMP treatment in the control siRNA. Whole-cell lysates were prepared and subjected to Western blotting to examine the knockdown of PGC-1α protein expression. β-Tubulin was used as an internal control. Immunoblot of β-tubulin is a reuse of Figure 5*A* because PGC-1α was examined in the same immunoblot membrane with C/EBPβ. The immunoblot is a representative of three different incubations. *C*, comparison of PGC-1α mRNA expression between nondecidualized ESCs (control), decidualized ESCs (cAMP), and HepG2 cells. PGC-1α mRNA expression was analyzed by quantitative real-time RT-PCR. Values of PGC-1α were normalized to those of MRPL19 and expressed as a ratio of the control of ESCs. SD of control samples is shown in Table S4. Values are mean ±SD of three different incubations. *A*, *p* < 0.01 *versus* control in ESCs. *D*, locations of the potential C/EBPβ-binding site in the enhancer regions of PGC-1α. For the ChIP assay, two regions were amplified by PCR: +8669 to +8823 bp and +25,280 to +25,395 bp for the PGC-1α enhancer regions. *E*, C/EBPβ recruitment to each enhancer region was examined by ChIP assay. Normal rabbit IgG was used as a negative control. The relative recruitment levels were analyzed by real-time PCR. Values were expressed as a ratio of control sample. SDs of control samples are shown in Table S4. Data are mean ±SD of three different incubations. *A*, *p* < 0.01 *versus* control treatment. *F*, effect of cAMP on the enhancer activities of the PGC-1α enhancer regions in ESCs. Each PGC-1α enhancer region was subcloned into pGL4.23 (enhancer/pGL4.23). ESCs were transfected with reporter vector (pGL4.23 or enhancer 1 or 2/pGL4.23) and pRL-TK vector as a normalization control. After 5 h of transfection, cells were treated in the presence or absence of cAMP for 4 days. The firefly luciferase activity was normalized according to Renilla luciferase activities. Values of the luciferase activities were expressed as a ratio of control sample with enhancer/pGL4.23. SDs of control samples are shown in Table S4. Values are mean ±SD of three different incubations. *A*, *p* < 0.01 *versus* control treatment sample with enhancer/pGL4.23. C/EBPβ, CCAAT/enhancer-binding protein beta; ChIP, chromatin immunoprecipitation; ESC, endometrial stromal cell; IgG, immunoglobulin G; IGFBP-1, insulin-like growth factor–binding protein-1; PGC-1α, peroxisome proliferator–activated receptor gamma coactivator 1 alpha.

Because we found that the upregulation of PGC-1α by cAMP is important for decidualization, we examined the detailed mechanisms by which PGC-1α is upregulated by cAMP. We focused on C/EBPβ as an upregulator of PGC-1α because C/EBPβ regulates many gene expressions during decidualization (4, 17, 40, 51). Moreover, it was reported that C/EBPβ regulates PGC-1α expression in regenerating the liver and differentiating adipocytes of mice (52, 53). Therefore, the effect of C/EBPβ knockdown on PGC-1α expression was examined. cAMP-increased C/EBPβ expressions were clearly suppressed by siRNA treatment (Fig. 5*A*). Knockdown of C/EBPβ decreased cAMP-increased PGC-1α mRNA and protein expressions (Fig. 5*B*). Furthermore, we examined the detailed mechanisms by which PGC-1α is upregulated by cAMP through C/EBPβ. For this purpose, we decided to investigate the genomic regions around PGC-1α to see whether potential C/EBPβ-binding sites exist. We first checked the public ChIP-sequence data of C/EBPβ bindings in HepG2 cells that are provided from the Encyclopedia of DNA Elements project (54). HepG2 is a human immortalized hepatocarcinoma cell line that expresses PGC-1α (55). We confirmed that HepG2 cells express PGC-1α mRNA as highly as decidualized ESCs (Fig. 5*C*). Therefore, C/EBPβ-binding peaks around the PGC-1α gene in HepG2 cells were searched. C/EBPβ-binding peaks were observed in the second intron of the PGC-1α gene, which are located approximately 8000 bp (enhancer region 1) and 25,000 bp (enhancer region 2) downstream from TSS. The consensus binding sequences of C/EBPβ were predicted by the JASPAR database (http://jaspar.binf.ku.dk/), and ChIP primers were designed for the regions surrounding the C/EBPβ consensus–binding sequences (enhancer region 1: +8609 bp to +8823 bp, enhancer region 2: +25,280 bp to +25.394 bp) (Fig. 5*D*). cAMP significantly increased the C/EBPβ recruitment to these two regions in ESCs (Fig. 5*E*). To determine whether the enhancer regions have enhancer activities, luciferase constructs containing the PGC-1α enhancer region (region 1/pGL4.23 or region 2/pGL4.23) were transfected into ESCs, respectively. cAMP increased luciferase activities of the PGC-1α enhancer regions, whereas no increases of luciferase activity were observed by cAMP in cells transfected with pGL4.23 alone (Fig. 5*F*). These results showed that both PGC-1α enhancer regions including C/EBPβ-binding sites have transcriptional activities by a decidualization stimulus.

### Effect of PGC-1α enhancer deletion by genome editing on cAMP-increased PGC-1α mRNA expression

**Figure 6:**
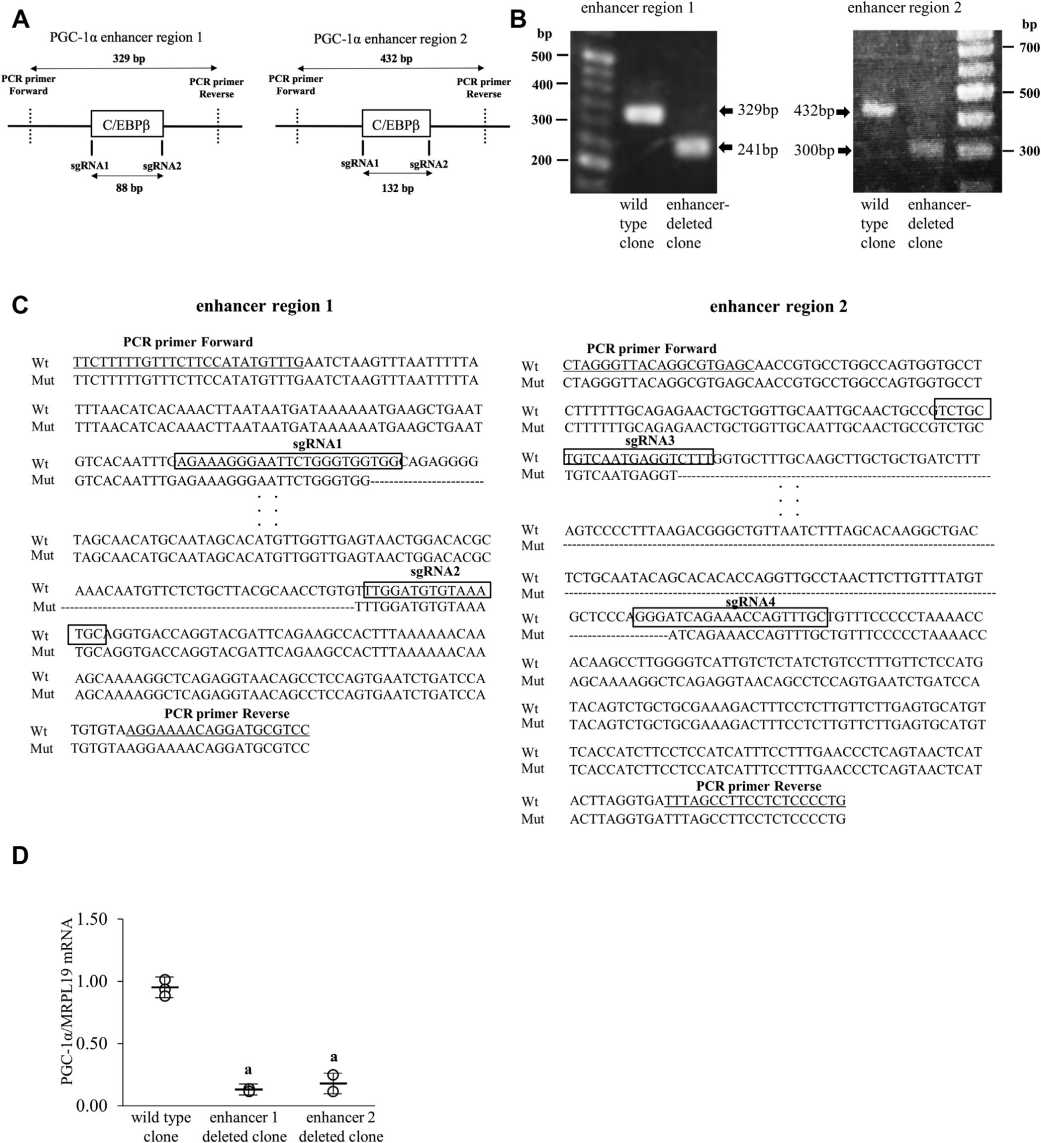
**Effect of PGC-1α enhancer deletion on cAMP-increased PGC-1α mRNA expression.***A*, the *vertical lines* represent the locations of sgRNA1 and sgRNA2 flanking the C/EBPβ-binding site in the PGC-1α enhancer regions. Two PCR primers surrounding two sgRNAs were used to amplify the genomic DNAs of each clone. *B*, PCR amplification products from genomic DNAs of the representative clones. For region 1 (*left*), a 329-bp PCR product was observed in wildtype clones. Successful deletion of the 88 bp generated between the two sgRNAs generated a smaller PCR product of 241 bp in enhancer region 1–deleted clones. For region 2 (*right*), a 432-bp PCR product was observed in wildtype clones. Successful deletion of the 132 bp generated between the two sgRNAs generated a smaller PCR product of 300 bp in enhancer region 1–deleted clones. *C*, DNA sequencing results around both deletion junctions amplified from genomic DNA of enhancer-deleted clone (Mut) (*left*; region 1, *right*; region 2). The deleted 88-bp and 132-bp regions are shown in *dashed lines*. The sequences of each sgRNA are boxed (sgRNA1 to sgRNA4). Primer sequences for genomic PCR are underlined. *D*, PGC-1α mRNA expression was analyzed by quantitative real-time RT-PCR. Values of PGC-1α were normalized to those of MRPL19 and expressed as a ratio of one wildtype sample. Values of each clone and mean ±SD are shown. *A*, *p* < 0.05 *versus*. wild type clone. C/EBPβ, CCAAT/enhancer-binding protein beta; PGC-1α, peroxisome proliferator–activated receptor gamma coactivator 1 alpha; sgRNA, single guide RNA.

Furthermore, to test the endogenous function of the PGC-1α enhancer regions on PGC-1α expression, we deleted these enhancer sequences from endogenous locus. We explored CRISPR/*CRISPR-*associated protein 9 (Cas9) technology to generate the cell lines that lack PGC-1α enhancer regions. Because of the low efficiency of transfection and difficulty of single cell culture for cloning, we could not generate proper ESC lines (data not shown). HepG2 cells, which highly express PGC-1α (Fig. 5*C*) and are widely used for the experiment on genome editing (21, 56) were used to examine the PGC-1α enhancer function by a genome editing approach. Single guide RNAs (sgRNAs) were designed to surround the C/EBPβ-binding sites in the two PGC-1α enhancer regions, respectively (Fig. 6*A*). HepG2 cells were cotransfected with CRISPR/Cas9 and sgRNA constructs. After cell cloning, the genomic DNAs of each clone were analyzed by PCR amplification with primers flanking two sgRNAs (Fig. 6*B*). For enhancer region 1, deleting the 88-bp region between the two sgRNAs would be expected to produce a 241-bp product, whereas a 329-bp product would be observed from genomic DNA in wildtype clones. For region 2, deleting the 132-bp region between the two sgRNAs would be expected to produce a 300-bp product, whereas a 432-bp product would be observed from genomic DNA in wildtype clones. Figure 6*B* shows the results of PCR amplification from genomic DNAs of representative wildtype and enhancer-deleted clones. Sequencing the DNA confirmed the deletion of the desired region in the enhancer-deleted clones (Fig. 6*C*). Clones that had the same deletion on both alleles were used for analysis. PGC-1α mRNA levels were analyzed and compared among wildtype clones and enhancer-deleted clones. Both enhancer region 1– and enhancer region 2–deleted clones showed significantly lower expressions of PGC-1α mRNA than the wildtype clones (Fig. 6*D*). These results indicated that the PGC-1α enhancer regions including the C/EBPβ-binding site are responsible for PGC-1α expression.

### Cellular functions regulated by PGC-1α during decidualization

**Figure 7:**
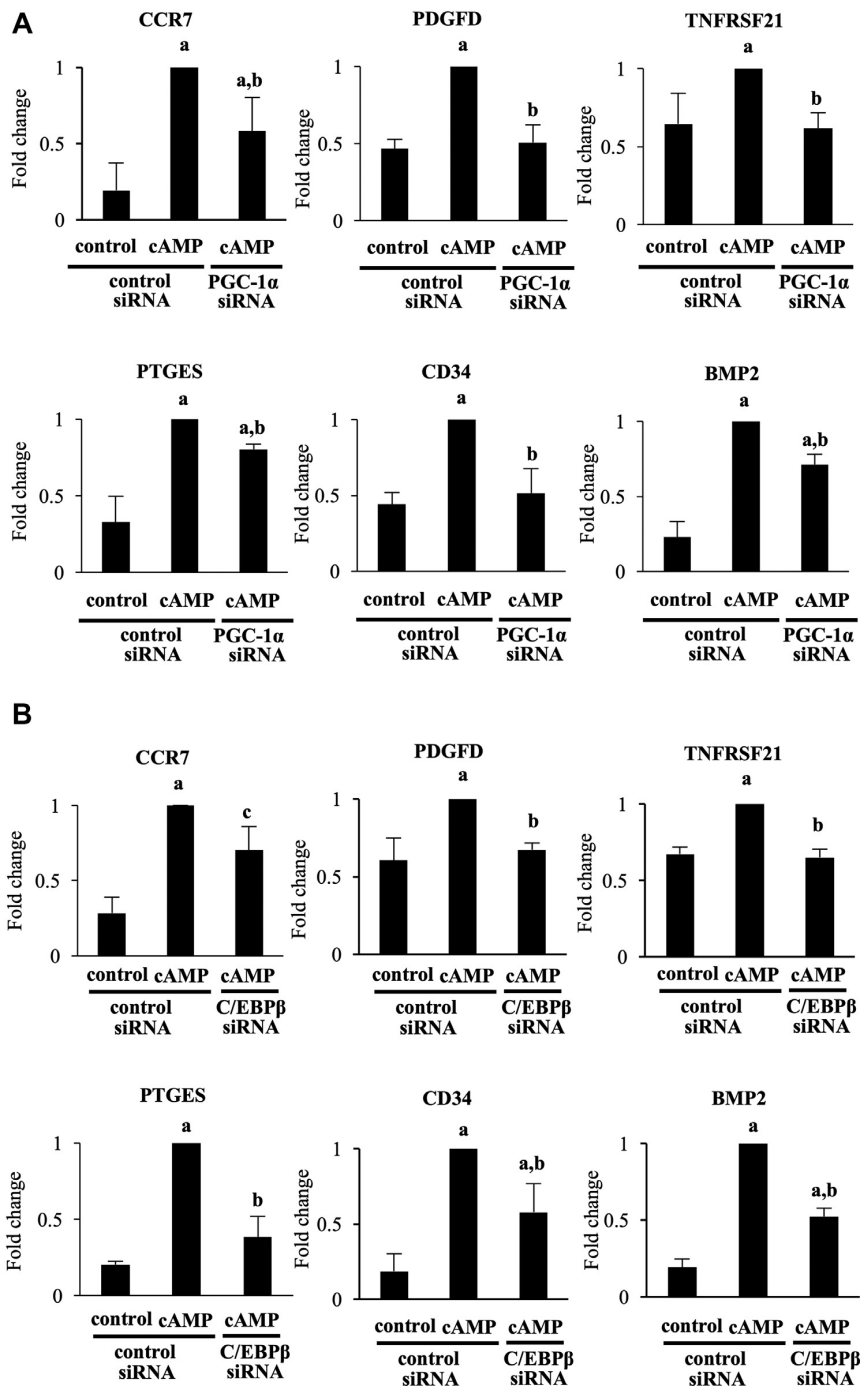
**Validation of RNA-sequence data by real-time RT-PCR.** ESCs were transfected with a siRNA targeted against PGC-1α (*A*), C/EBPβ (*B*), or with a nontargeting siRNA as a control. Forty-eight days after siRNA transfection, ESCs were treated with or without cAMP for 4 days. mRNA expression was analyzed by quantitative real-time RT-PCR. Values of PGC-1α–upregulated genes were normalized to those of MRPL19 and expressed as a ratio of the cAMP treatment sample in the control siRNA. SDs of cAMP treatment in the control siRNA sample are shown in Table S4. Values are mean ±SD of three different incubations. *A*, *p* < 0.01 *versus*. control treatment in the control siRNA; *B*, *p* < 0.01 *versus*. cAMP treatment in the control siRNA. C/EBPβ, CCAAT/enhancer-binding protein beta; ESC, endometrial stromal cell; PGC-1α, peroxisome proliferator–activated receptor gamma coactivator 1 alpha.

Last, to identify genes and cellular functions regulated by PGC-1α during decidualization, total RNAs were extracted from the three groups (no treatment with control siRNA, cAMP treatment with control siRNA, and cAMP treatment with PGC-1α siRNA) and subjected to RNA sequence. cAMP upregulated 2702 genes, 840 genes (31.1%) of which were suppressed by PGC-1α knockdown. They were designated as PGC-1α–upregulated genes during decidualization. The top 20 gene ontology terms for these 840 genes are shown in Table 1. In the PGC-1α–upregulated genes, a number of terms associated with immunomodulation (Table 1, shown in bold) were extracted. To validate the results of RNA sequence, we selected six PGC-1α upregulated genes associated with immunomodulation (CCR7, PDGFD, TNFRSF21, PTGES, CD34, and BMP2) for real-time RT-PCR analysis. cAMP increased their expressions, and knockdown of PGC-1α inhibited them (Fig. 7*A*). Furthermore, we examined whether these immunomodulation-related genes were also under the regulation of C/EBPβ. C/EBPβ knockdown inhibited their cAMP-increased expressions (Fig. 7*B*). These results suggested that C/EBPβ-PGC-1α pathway may be involved in immunomodulation during decidualization in addition to induction of decidualization. These results suggested that PGC-1α is involved in immunomodulation during decidualization in addition to induction of decidualization. On the other hand, cAMP downregulated 2819 genes, 361 genes (12.8%) of which were suppressed by PGC-1α knockdown. A gene ontology analysis of these genes showed no significantly enriched terms (data not shown).

**Table 1:** Enriched gene ontology terms associated with PGC-1α upregulated genes during decidualization

GO term	Genes	*p* value
**Positive regulation of leukocyte migration**	PDGFD, CCR7, LGALS9, ELANE, PF4V1	0.001991884
Positive regulation of protein kinase activity	NTRK1, GNG3, BMP2, PDGFD, CCR7, PILRB, LPAR3, FAM20A, ELANE	0.002066264
Positive regulation of kinase activity	NTRK1, GNG3, BMP2, PDGFD, CCR7, PILRB, LPAR3, FAM20A, ELANE	0.003214037
**Acute inflammatory response to antigenic stimulus**	NPFF, CCR7, ELANE	0.005107293
Cell–cell signaling	NTRK1, IGFBP1, BCHE, KCNIP2, NPFF, GPR21, LPAR3, OXT, AREG, BMP2, FRZB, WIF1, KCNMB3, CD34, CCL27, GRIA4	0.006215678
**Regulation of cytokine secretion**	CRTAM, CCR7, LGALS9, CD34, TNFRSF21	0.006240759
**Regulation of leukocyte migration**	PDGFD, CCR7, LGALS9, ELANE, PF4V1	0.007153459
**Leukocyte-mediated cytotoxicity**	RAET1G, CRTAM, LGALS9, ELANE	0.007533977
Response to inorganic substance	NTRK1, KCNIP2, PDGFD, MT1M, KCNMB3, CCR7, AREG, PTGES	0.007959356
**Cytokine secretion**	CRTAM, CCR7, LGALS9, CD34, TNFRSF21	0.008681458
Synaptic signaling	NTRK1, BCHE, KCNIP2, GPR21, NPFF, KCNMB3, OXT, LPAR3, GRIA4	0.009224994
Anterograde *trans*-synaptic signaling	NTRK1, BCHE, KCNIP2, GPR21, NPFF, KCNMB3, OXT, LPAR3, GRIA4	0.009224994
*Trans*-synaptic signaling	NTRK1, BCHE, KCNIP2, GPR21, NPFF, KCNMB3, OXT, LPAR3, GRIA4	0.009224994
Chemical synaptic transmission	NTRK1, BCHE, KCNIP2, GPR21, NPFF, KCNMB3, OXT, LPAR3, GRIA4	0.009224994
Positive regulation of MAPK cascade	NTRK1, GNG3, BMP2, PDGFD, CCR7, LPAR3, LGALS9, ELANE	0.009475397
Positive regulation of ERK1 and ERK2 cascade	NTRK1, BMP2, PDGFD, CCR7, LGALS9	0.011031014
Response to oxygen-containing compound	NTRK1, IGFBP1, BCHE, GPR21, OXT, AREG, GNG3, PDGFD, ID1, CCR7, LGALS9, ELANE, TNFRSF21, PF4V1, PTGES	0.011042849
Response to nitrogen compound	NTRK1, IGFBP1, BCHE, GNG3, PDGFD, GPR21, NPFF, ID1, CCR7, OXT, AREG	0.011628556
Regulation of ossification	BMP2, ID1, ID4, OXT, AREG	0.012119981
**Inflammatory response**	BMP2, NPFF, PLA2G4B, CCR7, LGALS9, ELANE, TNFRSF21, PF4V1, PTGES	0.012442969

## Discussion

**Figure 8:**
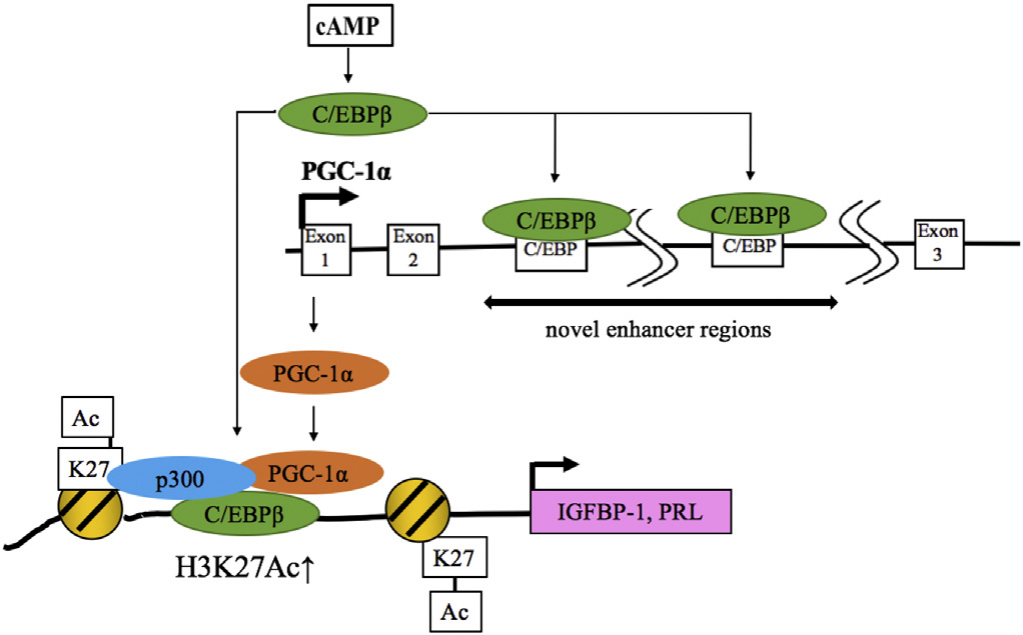
**Role and the regulation of PGC1α during decidualization.** PGC-1α is upregulated by the C/EBPβ recruitment to the novel enhancer regions during decidualization. PGC-1α contributes to decidualization by forming a histone-modifying complex with C/EBPβ and p300 and increasing H3K27ac in the promoter and enhancer regions of IGFBP-1 and the promoter region of PRL. C/EBPβ, CCAAT/enhancer-binding protein beta; ESC, endometrial stromal cell; H3K27ac, acetylation of histone-H3 lysine-27; IGFBP-1, insulin-like growth factor–binding protein-1; PGC-1α, peroxisome proliferator–activated receptor gamma coactivator 1 alpha; PRL, prolactin.

PGC-1α is well known as a transcription coactivator involved in mitochondrial biogenesis, thermogenesis, glucose metabolism, and fatty acid oxidation, especially in the liver, muscle, and adipose tissue (25, 26, 27, 28). PGC-1α also has physiological roles in the reproductive organs, such as the ovary and testis (29, 30, 31). Although the human endometrium is reported to express PGC-1α (32), the physiological functions of PGC-1α have been unclear. In this study, we found that PGC-1α works as a transcription coactivator that forms a histone-modifying complex with C/EBPβ and p300, induces epigenomic changes of IGFBP-1 and PRL, and contributes to decidualization. Furthermore, we revealed a novel regulatory mechanism for PGC-1α expression, in which C/EBPβ upregulates PGC1α expression by binding to novel enhancer regions (Fig. 8).

IGFBP-1 and PRL are induced in ESCs during decidualization and are therefore recognized as markers of decidualization (10, 15, 57). We and others previously identified several transcription factors that bind to their promoter regions, such as C/EBPβ, WT1, and FOXO1 (10, 17, 40, 42, 43, 44). We recently identified a novel enhancer region of IGFBP-1 that is approximately 5000 bp upstream from the TSS. Decidualization also stimulates C/EBPβ to bind there (21). However, there have been no reports showing the involvement of PGC-1α in the regulation of IGFBP-1 and PRL. Because PGC-1α does not have DNA-binding domains, it binds to the promoter and enhancer by interacting with other transcription factors (39, 58, 59, 60). The present study showed that decidualization induces the recruitment of PGC-1α to the C/EBPβ-binding sites in the promoter and enhancer regions of IGFBP-1 and the promoter region of PRL, suggesting that PGC-1α upregulates IGFBP-1 and PRL expression by interacting with C/EBPβ at their promoter and enhancer regions. In fact, our co-IP assay showed that PGC-1α interacts with C/EBPβ. Furthermore, we showed that C/EBPβ knockdown inhibited the recruitment of PGC-1α. This is also supported by the finding that PGC-1α interacts with C/EBPβ to activate target genes in human cancer cell lines (37, 38). Therefore, our study shows that PGC-1α works as a transcriptional coactivator of decidualization marker genes by cooperating with C/EBPβ.

We previously reported that the recruitment of C/EBPβ is a key event in H3K27ac in the promoter and enhancer regions of IGFBP-1 and the promoter region of PRL (17, 21). In other words, C/EBPβ works as a pioneer factor to initiate the chromatin remodeling of these regions. However, in order for pioneer factors to induce chromatin remodeling, they must form a histone-modifying complex with cofactors with HAT activities. We previously reported that p300 is a HAT that induces H3K27ac by cooperating with C/EBPβ at the IGFBP-1 enhancer region (21). The present study adds the new finding that decidualization also stimulates the recruitment of p300 to the C/EBPβ-binding sites in the promoter regions of IGFBP-1 and PRL. Therefore, p300 is an important HAT involved in the increase of H3K27ac in these three regions and following IGFBP-1 and PRL expression during decidualization. Furthermore, we showed that knockdown of PGC-1α suppressed the cAMP-increased p300 recruitment and H3K27ac levels in these three regions, indicating that PGC-1α contributes to the induction of H3K27ac by recruiting p300 into the histone-modifying complex. The histone-modifying complex contains not only a pioneer factor and HAT but also other coactivators (61). Although these coactivators themselves do not have HAT activities, they are involved in inducing histone acetylation by regulating the recruitment of HATs into the histone-modifying complex (62). Our idea is supported by the findings that PGC-1α has been reported to form a histone-modifying complex with p300 (33, 34) and to contribute to the induction of histone acetylation in kidney and liver cells (35, 36). In fact, our co-IP assay showed that p300 interacts with both PGC-1α and C/EBPβ. Taken together, these findings indicate that PGC-1α is a coactivator that forms a histone-modifying complex with C/EBPβ and p300 and is involved in the increase of H3K27ac of IGFBP-1 and PRL during decidualization. Although PGC-1α itself does not have HAT activities, it has an important role in the induction of epigenetic changes in ESCs. Unexpectedly, knockdown of PGC-1α reduced the C/EBPβ recruitment levels at the promoter and enhancer regions of IGFBP-1 and the promoter region of PRL. This was very likely due to the suppression of C/EBPβ recruitment to these regions by PGC-1α knockdown because knockdown of PGC-1α did not affect the C/EBPβ protein expression level. In other words, PGC-1α helps a pioneer factor to gain access to a genomic locus. This is not surprising because pioneer factors cannot bind to promoters in the absence of pairing cofactors (22). All cofactors forming a histone-modifying complex are essential so that pioneer factors can access the genomic locus and open the chromatin structure. However, we cannot completely exclude the possibility that other transcription factors than C/EBPβ work as pioneer factors. If the expressions of these pioneer factors were regulated by PGC-1α, PGC-1 α knockdown would downregulate them, which results in the decrease of C/EBPβ recruitment. In addition, we did not examine the interaction of PGC-1α with C/EBPβ or p300 on the promoter or enhancer regions. Further studies are needed to confirm their actual interactions on the genomic locus by performing assays, such as a sequential-ChIP assay (63).

Because PGC-1α is an important transcription factor for decidualization (Fig. 8), we further examined how decidualization increases PGC-1α expression. The present results clearly show that C/EBPβ upregulates PGC-1α expression during decidualization. So far, involvement of C/EBPβ in the regulation of PGC-1α expression has been observed in only mouse cells, in which C/EBPβ binds to the proximal promoter region of PGC-1α (52, 53). Although several transcription factor–binding sites have been identified in the proximal promoter region of PGC-1α (64), regions other than the proximal promoter have not been examined in the regulation of PGC-1α. Recently, not only the regions near TSS but also distal enhancer regions have been considered as important *cis*-elements for transcription because long-range chromatin interactions, such as enhancer–promoter interactions, can regulate gene expression levels (65, 66). Public ChIP-sequence data of C/EBPβ binding around the PGC-1α gene show two candidate C/EBPβ-binding sites in the enhancer regions (approximately 8000 bp and 25,000 bp downstream from the TSS). On the other hand, C/EBPβ-binding signals were not observed in the promoter regions. These observations suggest that, unlike mice cells, C/EBPβ regulates PGC-1α expression mainly through the binding to the enhancer regions rather than the promoter region in human cells. Therefore, we focused on the enhancer regions and found that C/EBPβ binds there during decidualization. The luciferase assay also revealed that these regions have transcriptional activities following a decidualization stimulus. However, so far, it has been difficult to validate the endogenous function of a putative enhancer. A reporter assay confirms that the DNA sequence of the enhancer region transfected exogenously has a transcriptional activity, but it does not demonstrate whether an adjacent gene can be activated by this enhancer *in vivo*. To overcome this problem, we used a CRISPR/Cas9 technique to test the endogenous function of the enhancer regions on PGC-1α expression. By establishing cell lines that lack enhancer regions by genome editing, we previously demonstrated the involvement of the distal enhancers in gene expressions (16, 40). In this study, by using a same technique, we clearly showed that the enhancer regions we focused on are involved in the PGC-1α expression. Therefore, they must be novel PGC-1α enhancers including novel C/EBPβ-binding sites. Because we used HepG2 cells, we cannot neglect the possibility that regulation of PGC-1α in HepG2 cells is different from that in decidualized ESCs. Taken together, our findings show that PGC-1α is upregulated by the C/EBPβ recruitment to novel enhancer regions during decidualization.

A related question is whether PGC-1α has functional roles other than contributing to decidualization. As suggested by the RNA sequence data, one possible role is regulating immune functions during decidualization. PGC-1α upregulates 840 genes during decidualization, indicating that PGC-1α is one of the essential transcription factors for decidualization. These genes appeared to be mainly associated with immunomodulation (Table 1). During decidualization, the endometrium undergoes a remarkable change of immune environment by the migration of immune cells (67, 68, 69). Although some reports indicated that PGC-1α is associated with the immunomodulation (70, 71), it has been unclear whether PGC-1α is involved in immunomodulation during decidualization. Among the PGC-1α–upregulated genes involved in immunomodulation, CCR7 is reported to regulate the migration of regulatory T cells into decidua (72). CD34 is one of the markers for the progenitor cells of decidual natural killer cells (73). Interestingly, these immunomodulation-associated genes upregulated by PGC-1α were also under the regulation of C/EBPβ. These results suggested that C/EBPβ–PGC-1α pathway may be involved in immunomodulation during decidualization. One may be wondering why PGC-1α does not influence the morphological change that accompanies decidualization, while it upregulates the expressions of IGFBP-1 and PRL, decidualization markers. A number of cellular functions alter with morphological change during decidualization, and these changes are cooperatively regulated by various key molecules, including transcription factors or transcriptional coactivators. Thus, each key molecule has a specific role for decidualization. All key molecules do not always regulate cellular morphology during decidualization. It is, therefore, likely that PGC-1α has a role for immunomodulation rather than morphological change during decidualization.

In summary, PGC-1α has a novel role in the human endometrium, in which PGC-1α contributes to decidualization by forming a histone-modifying complex with C/EBPβ and p300 and inducing epigenomic changes of IGFBP-1 and PRL. Moreover, our results show that PGC-1α is upregulated by the recruitment of C/EBPβ to novel enhancer regions and that a C/EBPβ–PGC-1α pathway plays an important role in decidualization in human ESCs.

## Experimental procedures

### Reagents

Dulbecco's modified Eagle's medium (DMEM), L-glutamine, 1X trypsin–EDTA, streptomycin, and penicillin were purchased from Invitrogen. Fetal bovine serum (FBS) was obtained from Biological Industries Ltd. Collagenases and dibutyryl-cAMP were obtained from Sigma Chemical Co Ltd. Tissue flasks were from Becton Dickinson Co Ltd.

### ESC isolation

Human endometrial tissues were obtained at hysterectomy from patients with a normal menstrual cycle, aged 40 to 45 years, who underwent surgery for myoma uteri or early-stage cervical cancer. The patients were not on hormonal therapy at the time of surgery. Informed consent was obtained from all participating patients, and ethical approval was obtained from the Institutional Review Board of Yamaguchi University Hospital. All experiments were performed in accordance with the Tenets of the Declaration of Helsinki. Endometrial samples utilized for ESC isolation were histologically diagnosed as being in the late proliferative phase according to published criteria (74). Tissue samples were washed with phenol red–free DMEM containing 4 mM glutamine, 50 μg/ml streptomycin, and 50 IU/ml penicillin and minced into pieces of <1 mm^3^. ESCs were isolated as reported previously (9). In brief, tissues were minced, enzymatically digested with 0.2% collagenase in a shaking water bath for 2 h at 37 °C, and filtered through a 70-μm nylon mesh. Stromal cells in the filtrates were washed three times with the medium, and the number of viable cells was counted by trypan blue dye exclusion. Under the microscope, all of the cells reacted with the stromal-reacting antibody vimentin (data not shown), indicating that they were homogeneous. The cells were also verified to be negative for an epithelial cell–reacting antibody (cytokeratin) (data not shown). Cells were seeded at 10^5^ cells/cm^2^ in 75-cm^2^ tissue culture flasks and incubated in phenol red–free DMEM containing glutamine, antibiotics, and 10% dextran-coated charcoal-stripped FBS at 37 °C, 95% air, and 5% CO_2_. At confluence, cells were treated with 1X trypsin–EDTA and subcultured into 6-well plates. At 80% confluence after the first passage, the cell culture medium was changed to the treatment medium.

### Cell culture

HepG2 cells were seeded in 75-cm^2^ tissue culture flasks and incubated in phenol red–free DMEM containing glutamine, antibiotics, and 10% dextran-coated charcoal-stripped FBS at 37 °C, 95% air, and 5% CO_2_. At confluence, cells were treated with 1X trypsin–EDTA and subcultured into 6-well plates and used for real-time RT-PCR and genome editing as described later. To induce decidualization, ESCs were incubated with treatment medium (phenol red–free DMEM supplemented with glutamine, antibiotics, and 2% dextran-coated charcoal-stripped FBS) containing cAMP (0.5 mM) for 4 days. The cells were then used for the experiments described later. In the normal menstrual cycle, progesterone induces decidualization of endometrial stromal cells. cAMP is a second messenger of progesterone (75). Because cAMP more clearly induces decidualization in a short time *in vitro* than progesterone does, it has been widely used as a decidualization stimulus (15). Therefore, we used cAMP as a decidualization inducer in this study. The concentration of cAMP (0.5 mM) and the period of incubation (4 days) used in this study were based on our previous report (75). The medium was changed every other day. Cells isolated from one patient were incubated one time in triplicate. Cells from at least three individuals were used for each experiment as we reported previously (76), except for RNA sequence.

### Immunohistochemistry

Tissue samples from the late proliferative phase, late secretory phase, and decidua of early pregnancy were immunostained as reported previously (17). The endometrial tissues were fixed in formalin, embedded in paraffin, and cut into 5-μm sections. The sections were deparaffinized in xylene, dehydrated in a graded series of ethanol, stained with Histofine simple stain MAX-PO(R) (Nichirei Co Ltd) using a rabbit polyclonal antibody to PGC-1α (Abcam plc), incubated in 3, 3′-diaminobenzidine-4 HCl (Nakalai Tesque Co Ltd) in 0.05 M Tris-HCl buffer (pH 7.6) containing 0.01% H_2_O_2_ for 3 min to visualize peroxidase activity, and counterstained with Meyer’s hematoxylin. Control sections were incubated with normal rabbit serum.

### Real-time RT-PCR

Total RNA was isolated from the cultured cells with an RNeasy Mini Kit (Qiagen). The RNA was reverse transcribed as reported previously (77). For PCR amplification, first strand complementary DNA was synthesized from 1 μg of total RNA with reverse transcriptase (Invitrogen) in 20 μl of reaction mixture. The relative mRNA expressions were examined by real-time RT-PCR as reported previously (78, 79) with sequence-specific primer sets (Table S1). MRPL19 was used as an internal control.

### Western blotting

Western blotting was performed as reported previously (80, 81). In brief, whole-cell lysates were prepared using loading buffer reagents (Santa Cruz Biotechnology, Inc) without trypsin treatment. Equal amounts of total protein were electrophoresed on a 10% SDS–polyacrylamide gel. The proteins were transferred to polyvinylidene difluoride membranes (ATTO). The membranes were blocked with blocking solution (5% skimmed milk with 0.1% Tween-20 dissolved in Tris-buffered saline [pH 7.5]), incubated with the first antibody for PGC-1α (Calbiochem), C/EBPβ (Santa Cruz Biotechnology), and β-tubulin (Sigma), which were diluted in blocking solution, incubated with the peroxidase-conjugated second antibody diluted in blocking solution, visualized with the ECL-Western blotting detection system (Amersham) according to the manufacturer's protocol, and used to expose hyperfilm-ECL (Amersham). To reuse the blot, the membranes were stripped in Restore Western stripping buffer (Pierce). Western blot bands were quantified by ImageJ (U. S. National Institutes of Health), and the quantification values of three independent experiments are provided in Table S2 of the revised manuscript. The uncropped images of immunoblots are shown in Fig. S1.

### Co-IP assay

The co-IP assay involving PGC-1α, C/EBPβ, and p300 was performed using a Capturem IP & Co-IP Kit (TaKaRa) according to the manufacturer’s protocol. In brief, cultured cells were lysed with 200 μl of lysis/equilibration buffer per 1 × 10^6^ cells and incubated on ice for 15 min; 10% of the cell lysate was kept as input. For immunoprecipitation, equal amount of proteins of cell lysates were incubated with the antibodies for PGC-1α antibody (Calbiochem), C/EBPβ (Santa Cruz Biotechnology), p300 (Abcam), and normal rabbit immunoglobulin G (Invitrogen) for 20 min at room temperature and then subjected to Western blotting with these antibodies, as described previously. Western blot bands were quantified by ImageJ, and the quantification values of three independent experiments are shown in Table S2 of the revised manuscript. The uncropped images of immunoblots are shown in Fig. S1.

### Lipid-mediated transfection of siRNA duplexes

PGC-1α ON-TARGET plus SMART pool, C/EBPβ ON-TARGET plus SMART pool, and ON-TARGET plus Non-Targeting pool siRNA were purchased from Dharmacon. ESCs at 50% confluence were transfected with siRNA duplexes (20 nM) and RNAi MAX (Invitrogen) as we reported previously (16). The medium was changed 5 h later. After 48 h of transfection, cells were incubated in the presence or absence of cAMP for 4 days and then used for the experiments described later.

### ChIP assay

The levels of transcription factor recruitment and H3K27ac levels were examined by the ChIP assay according to the protocol for the ChIP assay kit (Upstate Biotechnology) as we reported previously (16, 21). Cells were cross-linked by the addition of formaldehyde into the medium at a final concentration of 1% and incubated for 10 min at 37 °C. Cross-linking was terminated by the addition of glycine (0.125 M, final concentration). Cells lysates were sonicated using a Bioruptor ultrasonicator (Cosmo Bio), precleared with salmon sperm DNA–protein A at 4 °C for 4 h. Five percent of the supernatants were kept as input controls (INPUT). Dynabeads Protein A (Invitrogen) were incubated with antibodies for PGC-1α (Novus Biologicals), C/EBPβ (Santa Cruz Biotechnology), p300 (Abcam), H3K27ac (generous gift from Dr H. Kimura) (82), and normal mouse or rabbit immunoglobulin G (Invitrogen) 4 °C overnight. The precleared chromatin was incubated with antibody-bound Dynabeads for 6 h at 4 °C to collect the immune complexes. The cross-linking of the immunoprecipitated chromatin complex and input control (INPUT; 2% of the total soluble chromatin) were reversed by heating the samples at 65 °C overnight and then subjected to proteinase K treatment. The DNA was purified using a QIAquick PCR purification kit (QIAGEN) and used as a template for PCR amplification with various primer sets (Table S1) to amplify specific regions. Real-time PCR was used to determine the relative levels of PGC-1α, C/EBPβ and p300 recruitment, and H3K27ac levels of each target sequence. The ratio of immunoprecipitated DNA to the INPUT DNA sample (%INPUT) was calculated as reported previously (21).

### Whole-transcriptome analysis with RNA sequence

ESCs from one patient were transfected with a siRNA targeted against PGC-1α or with a nontargeting siRNA as a control. Forty-eight hours after siRNA transfection, ESCs were treated with or without cAMP for 4 days. Total RNAs extracted from three samples (control siRNA with no treatment, control siRNA with cAMP treatment, and WT1 siRNA with cAMP treatment) were subjected to RNA-sequence analysis as reported previously (4, 7, 83). mRNA was purified with oligo dT beads (NEBNext Poly(A) mRNA magnet Isolation Module, New England Biolabs). Complementary DNA libraries for Illumina sequencing were generated with NEBNext Ultra II RNA library Prep kit (NEB) and NEBNextplex Oligos for Illumina. The confirmed libraries were sequenced with Illumina Next-seq DNA sequencer with a 75-bp pair-end cycle sequencing kit (Illumina). To produce the raw bcl, or base call files, quality assessment and image analyses were performed using Next-seq packaging software (Illumina) Real Time Analysis, and bcl2fastaq Conversion Software v2.19 (Illumina) was used for demultiplexing of the samples. Reads with more than two ambiguous nucleotides and reads with quality scores less than 20 as calculated by the Phred program were removed using CLC Genomics Workbench software (version 8.01, QIAGEN). Long reads with more than 1000 nucleotides and short reads with fewer than 20 nucleotides were also discarded. Trimmed reads were mapped to the human reference genome GRCh38 in default settings. Gene expression values were calculated as “Reads Per Kilobase of exon per Million mapped reads.” We added 1 to the Reads Per Kilobase of exon per Million mapped reads value before the following calculation. When gene expression levels differed by more than 1.5-fold between two samples, they were defined as differentially expressed genes. DAVID Bioinformatics Resources, version 6.8 (https://david.ncifcrf.gov/), was used to determine whether the functional annotation of the differentially expressed genes was enriched for specific gene ontology terms (84). *p*-values < 0.05 were considered to indicate significant enrichment.

### Luciferase assay

Two PGC-1α enhancer regions were amplified by PCR from the human genomic DNA. The PCR products were subcloned upstream of the luciferase gene into firefly luciferase vectors pGL4.23 (Promega), which contains minimal promoter. The constructs were termed enhancer/pGL4.23. ESCs were cultured on a 24-well plate (5 × 10^4^ cells/well) for 24 h and then transfected with reporter vector (pGL4.23-basic or enhancer/pGL4.23) and pRL-TK vector (Promega) as a normalization control using Lipofectamine LTX (Invitrogen). After 24 h of transfection, cells were treated in the presence or absence of cAMP for 4 days. The firefly and Renilla luciferase activities were measured using a Dual-Luciferase Reporter Assay System (Promega) according to the manufacturer’s instructions.

### Deletion of the PGC-1α enhancer region by CRISPR/Cas9 system

pCAGmCherry-gRNA (Addgene plasmid # 87110) was a gift from Dr Juan Carlos Izpisua Belmonte (The Salk Institute). pCas9_GFP was a gift from Kiran Musunuru (Addgene plasmid # 44719). To construct sgRNA expression vectors, each 20-bp target sequence was subcloned into pCAGmCherry-gRNA vector. The CRISPR/Cas9 target sequences used in this study are sgRNA1: AGAAAGGGAATTCTGGGTGGTGG; sgRNA2: GCATTTACACATCCAAACACAGG; sgRNA3: TCTGCTGTCAATGAG GTCTTTGG; and sgRNA4: GCAAACTGGTTTCTGAT CCCTGG, in which the 3-bp protospacer adjacent motif sequences are underlined (Fig. 6*C*). HepG2 cells were plated in medium lacking antibiotics at approximately 3 × 10^5^ cells in a 6-well plate 1 day before transfection. At 50% confluence, Cas9 plasmid (1 μg) and two guide RNA plasmids (0.5 μg each) were transfected to HepG2 cells with Lipofectamine 3000 (Invitrogen). After 48 h of transfection, cells were trypsinized, diluted, and plated in 6-well plates. After 10 days, individual colonies were isolated, harvested, seeded in 24-well plates, and grown until confluence when they were transferred to 6-well plates. To screen the enhancer deletion, the genomic DNAs of each clone were analyzed by PCR amplification with primers flanking the two sgRNAs. Twenty nanograms of genomic DNA was used and amplified with PrimeSTAR GXL DNA Polymerase (TaKaRa). The resulting products were subjected to agarose gel electrophoresis and purified using a QIAquick gel extraction kit (QIAGEN). The PCR products were cloned into pGEM-T easy vector (Promega), and sequencing was performed using an ABI automated sequencer with BigDye terminators (Applied Biosystems). Three wildtype clones, three enhancer 1–deleted clones, and three enhancer 2–deleted clones were used as representative clones. PGC-1α mRNA expressions were analyzed in each clones and were expressed as the means of three clones.

### Statistical analysis

Statistical significance was determined by one-way ANOVA. After ANOVA, the Tukey–Kramer test was applied to analyze differences between groups. An unpaired *t* test was applied to analyze the difference between two groups. All statistical analyses were performed using SPSS for Windows, version 11 (SPSS Inc). Differences were considered significant at *p* < 0.05. The exact *p* values in each statistical analysis are provided in Table S3.

## Data availability

RNA-seq data are deposited in Gene Expression Omnibus (GEO) (GEO accession no.: GSE167990).

## Supporting information

This article contains supporting information Fig. S1 and Tables S1–S4.

## Conflict of interest

The authors declare that they have no conflicts of interest with the contents of this article.
