# Supplementary material for: Transcriptional coactivator PGC-1α contributes to decidualization by forming a histone-modifying complex with C/EBPβ and p300
Source: J Biol Chem. 2022 Mar 28;298(5):101874. doi: 10.1016/j.jbc.2022.101874 (PMC9048111; doi:10.1016/j.jbc.2022.101874)
Supplement: Supplemental Tables S1–S4 and Figure S1 [file mmc1.pdf]

Supplemental material to

Transcriptional coactivator PGC-1α contributes to decidualization by forming a histone-modifying complex with C/EBPβ and p300

Haruka Takagi, Isao Tamura*, Taishi Fujimura, Yumiko Doi-Tanaka, Yuichiro Shirafuta,
Yumiko Mihara, Ryo Maekawa, Toshiaki Taketani, Shun Sato, Hiroshi Tamura, Norihiro Sugino

Including
Table S1-S4
Figure S1

Table S1. Primer sequences used in this study

ChIP-qPCR	Forward	Reverse
IGFBP-1 promoter	GCCTGAACCCCCTAACAAC	TTGCACCAGGAGGTTAATGA
IGFBP-1 enhancer (for H3K27ac)	CATCGCCAACACTGAGATTC	CTCTTGTCACGTCAACAAAACC
IGFBP-1 enhancer (for transcription factor recruitment)	TTTTCCCCGTGTTAAAAACAAC	TCACCAGCAGCTGAAAATTG
PRL promoter	AGCAGCACTACTGACATTTGGA	GTGTTCTGAACCCCATCAATCT
PGC-1α enhancer1	GAGGGGTAGCAACATGCAAT	CCTCTGAGCCTTTTGCTTT
PGC-1α enhancer2	CCCTTTAAGACGGGCTGTTA	CAGCAAACTGGTTTCTGATCC

Real-time RT-PCR	Forward	Reverse
PGC-1α	GCTTTCTGGGTGGACTCAAGT	GAGGGCAATCCGTCTTCATCC
IGFBP-1	CGAAGGCTCTCCATGTCACCA	TGTCTCCTGTGCCTTGGCTAAAC
PRL	AAAGGATCGCCATGGAAAG	GCACAGGAGCAGGTTTGAC
CCR7	TGGTGGTGGCTCTCCTTG	GTTCCGCACGTCCTTCTTG
PDGFD	GTGGAGGAAATTGTGGCTGT	CGTTCATGGTGATCCAACTG
TNFRSF21	AGCACCGGAGAAACGATGTT	GGGCTCATCGGGAGAGCTA
PTGES	GGAGACCATCTACCCCTTCCT	AAGTGCATCCAGGCGACAAA
CD34	TCCCAAAAGACCCTGATTGC	CTCCACCGTTTTCCGTGTAA
BMP2	CCACCATGAAGAATCTTTGGAAGAAC	TGATAAACTCCTCCGTGGGGA
MRPL19	GAATGTTATCGAAGGACAAGGT	CAGGAAGGGCATCTCGTAAG

Table S2. Quantification data of immunoblots in three independent experiments

Fig. 1	B	IB-PGC-1α	control	cAMP	
		rep .1	1.00	2.64	
		rep .2	1.00	2.94	
		rep .3	1.00	3.07	
Fig. 2	A	IB-PGC-1α	control siRNA-control	control siRNA-cAMP	PGC-1α siRNA-cAMP
		rep .1	1.00	2.08	1.36
		rep .2	1.00	3.18	1.13
		rep .3	1.00	4.14	1.75
Fig. 3	B	IB-C/EBPβ	INPUT	control	cAMP
			rep .1	1.00	1.95
			rep .2	1.00	1.79
			rep .3	1.00	2.51
			IP-PGC-1α	control	cAMP
			rep .1	1.00	1.67
			rep .2	1.00	3.58
			rep .3	1.00	1.76
			IP-C/EBPβ	control	cAMP
			rep .1	1.00	1.75
			rep .2	1.00	2.49
			rep .3	1.00	3.32
			IP-p300	control	cAMP
			rep .1	1.00	2.64
			rep .2	1.00	3.34
			rep .3	1.00	3.12
		IB-PGC-1α	INPUT	control	cAMP
			rep .1	1.00	2.65
			rep .2	1.00	2.38
			rep .3	1.00	2.94
			IP-PGC-1α	control	cAMP
			rep .1	1.00	2.45
			rep .2	1.00	4.61
			rep .3	1.00	3.45

		IP-C/EBPβ	control	cAMP
		rep .1	1.00	4.66
		rep .2	1.00	7.02
		rep .3	1.00	5.44

		IP-p300	control	cAMP
		rep .1	1.00	2.36
		rep .2	1.00	1.53
		rep .3	1.00	1.59

Fig. 4	F	IB-PGC-1α	control siRNA-control	control siRNA-cAMP	PGC-1α siRNA-cAMP
		rep .1	1.00	2.57	1.11
		rep .2	1.00	4.62	2.01
		rep .3	1.00	3.51	1.49
		IB-C/EBPβ	control siRNA-control	control siRNA-cAMP	PGC-1α siRNA-cAMP
		rep .1	1.00	2.39	2.19
		rep .2	1.00	2.62	2.29
		rep .3	1.00	2.68	2.70

Fig. 5	A	IB-C/EBPβ	control siRNA-control	control siRNA-cAMP	C/EBPβ siRNA-cAMP
		rep .1	1.00	1.83	0.48
		rep .2	1.00	2.03	0.49
		rep .3	1.00	1.88	0.57
	B	IB-PGC-1α	control siRNA-control	control siRNA-cAMP	C/EBPβ siRNA-cAMP
		rep .1	1.00	3.81	1.94
		rep .2	1.00	2.68	1.46
		rep .3	1.00	2.07	1.11

Table S3. P values in each statistical analysis

Fig. 1	B	PGC-1α	control. vs cAMP.	p=0.007
		IGFBP-1	control. vs cAMP.	p=0.00078
		PRL	control. vs cAMP.	p=0.00094
Fig. 2	B	IGFBP-1	control (control siRNA) vs. cAMP (control siRNA)	p=0.000002
			control (control siRNA) vs. cAMP (PGC-1α siRNA)	p=0.000217
			cAMP (control siRNA) vs. cAMP (PGC-1α siRNA)	p=0.00008
		PRL	control (control siRNA) vs. cAMP (control siRNA)	p=0.00000024868
			control (control siRNA) vs. cAMP (PGC-1α siRNA)	p=0.00000025106
			cAMP (control siRNA) vs. cAMP (PGC-1α siRNA)	p=0.00000024879
Fig. 3	C	IGFBP-1 promoter	control. vs cAMP.	p=0.0085
		IGFBP-1 enhancer	control. vs cAMP.	p=0.043
		PRL promoter	control. vs cAMP.	p=0.0089
	D	IGFBP-1 promoter	control. vs cAMP.	p=0.0097
		IGFBP-1 enhancer	control. vs cAMP.	p=0.007
		PRL promoter	control. vs cAMP.	p=0.0095
Fig. 4	A	IGFBP-1 promoter	control (control siRNA) vs. cAMP (control siRNA)	p=0.013
			cAMP (control siRNA) vs. cAMP (PGC-1α siRNA)	p=0.041
		IGFBP-1 enhancer	control (control siRNA) vs. cAMP (control siRNA)	p=0.004
			cAMP (control siRNA) vs. cAMP (PGC-1α siRNA)	p=0.004
		PRL promoter	control (control siRNA) vs. cAMP (control siRNA)	p=0.000042
			cAMP (control siRNA) vs. cAMP (PGC-1α siRNA)	p=0.000154
	B	IGFBP-1 promoter	control (control siRNA) vs. cAMP (control siRNA)	p=0.001
			cAMP (control siRNA) vs. cAMP (PGC-1α siRNA)	p=0.003
		IGFBP-1 enhancer	control (control siRNA) vs. cAMP (control siRNA)	p=0.001
			cAMP (control siRNA) vs. cAMP (PGC-1α siRNA)	p=0.006
		PRL promoter	control (control siRNA) vs. cAMP (control siRNA)	p=0.002
			cAMP (control siRNA) vs. cAMP (PGC-1α siRNA)	p=0.022
	C	IGFBP-1 promoter	control (control siRNA) vs. cAMP (control siRNA)	p=0.000119
			cAMP (control siRNA) vs. cAMP (C/EBPβ siRNA)	p=0.00046
		IGFBP-1 enhancer	control (control siRNA) vs. cAMP (control siRNA)	p=0.011
			cAMP (control siRNA) vs. cAMP (C/EBPβ siRNA)	p=0.024
		PRL promoter	control (control siRNA) vs. cAMP (control siRNA)	p=0.000194
			cAMP (control siRNA) vs. cAMP (C/EBPβ siRNA)	p=0.000251

	D	IGFBP-1 promoter	control (control siRNA) vs. cAMP (control siRNA)	p=0.000049
			cAMP (control siRNA) vs. cAMP (C/EBPβ siRNA)	p=0.000125
		IGFBP-1 enhancer	control (control siRNA) vs. cAMP (control siRNA)	p=0.001
			cAMP (control siRNA) vs. cAMP (C/EBPβ siRNA)	p=0.001
		PRL promoter	control (control siRNA) vs. cAMP (control siRNA)	p=0.000016
			cAMP (control siRNA) vs. cAMP (C/EBPβ siRNA)	p=0.000016
	E	IGFBP-1 promoter	control (control siRNA) vs. cAMP (control siRNA)	p=0.007
			cAMP (control siRNA) vs. cAMP (PGC-1α siRNA)	p=0.025
		IGFBP-1 enhancer	control (control siRNA) vs. cAMP (control siRNA)	p=0.005
			cAMP (control siRNA) vs. cAMP (PGC-1α siRNA)	p=0.039
		PRL promoter	control (control siRNA) vs. cAMP (control siRNA)	p=0.002
			cAMP (control siRNA) vs. cAMP (PGC-1α siRNA)	p=0.027
Fig. 5	B	PGC-1α	control (control siRNA) vs. cAMP (control siRNA)	p=0.003
			cAMP (control siRNA) vs. cAMP (C/EBPβ siRNA)	p=0.046
	C	PGC-1α	control (ESC) vs. cAMP (ESC)	p=0.000424
			control (ESC) vs. control (HepG2)	p=0.000269
	E	enhancer1	control. vs cAMP.	p=0.007
		enhancer2	control. vs cAMP.	p=0.004
	F	enhancer1	control. vs cAMP.	p=0.0048
		enhancer2	control. vs cAMP.	p=0.000404
Fig. 6	D	PGC-1α	WT vs deletion lesion1	p=0.000016
			WT vs deletion lesion2	p=0.000034
Fig. 7	A	CCR7	control (control siRNA) vs. cAMP (control siRNA)	p=0.000478
			control (control siRNA) vs. cAMP (PGC-1α siRNA)	p=0.008
			cAMP (control siRNA) vs. cAMP (PGC-1α siRNA)	p=0.006
		PDGFD	control (control siRNA) vs. cAMP (control siRNA)	p=0.00031
			cAMP (control siRNA) vs. cAMP (PGC-1α siRNA)	p=0.000488
		TNFRSF21	control (control siRNA) vs. cAMP (control siRNA)	p=0.003
			cAMP (control siRNA) vs. cAMP (PGC-1α siRNA)	p=0.0024
		PTGES	control (control siRNA) vs. cAMP (control siRNA)	p=0.00039
			control (control siRNA) vs. cAMP (PGC-1α siRNA)	p=0.003
			cAMP (control siRNA) vs. cAMP (PGC-1α siRNA)	p=0.009
		CD34	control (control siRNA) vs. cAMP (control siRNA)	p=0.001
			cAMP (control siRNA) vs. cAMP (PGC-1α siRNA)	p=0.003
		BMP2	control (control siRNA) vs. cAMP (control siRNA)	p=0.000029

		control (control siRNA) vs. cAMP (PGC-1α siRNA)	p=0.006
		cAMP (control siRNA) vs. cAMP (PGC-1α siRNA)	p=0.006
B	CCR7	control (control siRNA) vs. cAMP (control siRNA)	p=0.002
		cAMP (control siRNA) vs. cAMP (C/EBPβ siRNA)	p=0.048
	PDGFD	control (control siRNA) vs. cAMP (control siRNA)	p=0.003
		cAMP (control siRNA) vs. cAMP (C/EBPβ siRNA)	p=0.009
	TNFRSF21	control (control siRNA) vs. cAMP (control siRNA)	p=0.000148
		cAMP (control siRNA) vs. cAMP (C/EBPβ siRNA)	p=0.000102
	PTGES	control (control siRNA) vs. cAMP (control siRNA)	p=0.003
		cAMP (control siRNA) vs. cAMP (C/EBPβ siRNA)	p=0.009
	CD34	control (control siRNA) vs. cAMP (control siRNA)	p=0.001
		control (control siRNA) vs. cAMP (C/EBPβ siRNA)	p=0.022
		cAMP (control siRNA) vs. cAMP (C/EBPβ siRNA)	p=0.016
	BMP2	control (control siRNA) vs. cAMP (control siRNA)	p=0.000001
		control (control siRNA) vs. cAMP (C/EBPβ siRNA)	p=0.000237
		cAMP (control siRNA) vs. cAMP (C/EBPβ siRNA)	p=0.00003

Table S4. SD of the control samples

		gene or region	SD of controls
Fig. 1	B	PGC-1α	0.06
		IGFBP-1	0.14
		PRL	0.12
Fig. 2	B	IGFBP-1	0.07
		PRL	0.08
Fig. 3	C	IGFBP-1 promoter	0.10
		IGFBP-1 enhancer	0.13
		PRL promoter	0.09
	D	IGFBP-1 promoter	0.18
		IGFBP-1 enhancer	0.14
		PRL promoter	0.13
Fig. 4	A	IGFBP-1 promoter	0.11
		IGFBP-1 enhancer	0.10
		PRL promoter	0.14
	B	IGFBP-1 promoter	0.07
		IGFBP-1 enhancer	0.13
		PRL promoter	0.10
	C	IGFBP-1 promoter	0.15
		IGFBP-1 enhancer	0.14
		PRL promoter	0.09
	D	IGFBP-1 promoter	0.13
		IGFBP-1 enhancer	0.08
		PRL promoter	0.12
	E	IGFBP-1 promoter	0.12
		IGFBP-1 enhancer	0.08
		PRL promoter	0.12

Fig. 5	B	PGC-1α	0.13
	C	PGC-1α	0.06
	E	enhancer1	0.09
		enhancer2	0.19
	F	enhancer1	0.15
		enhancer2	0.16
Fig. 6	D	PGC-1α	0.11
Fig. 7	A	CCR7	0.12
		PDGFD	0.08
		TNFRSF21	0.10
		PTGES	0.09
		CD34	0.11
		BMP2	0.11
	B	CCR7	0.10
		PDGFD	0.12
		TNFRSF21	0.09
		PTGES	0.12
		CD34	0.10
		BMP2	0.12

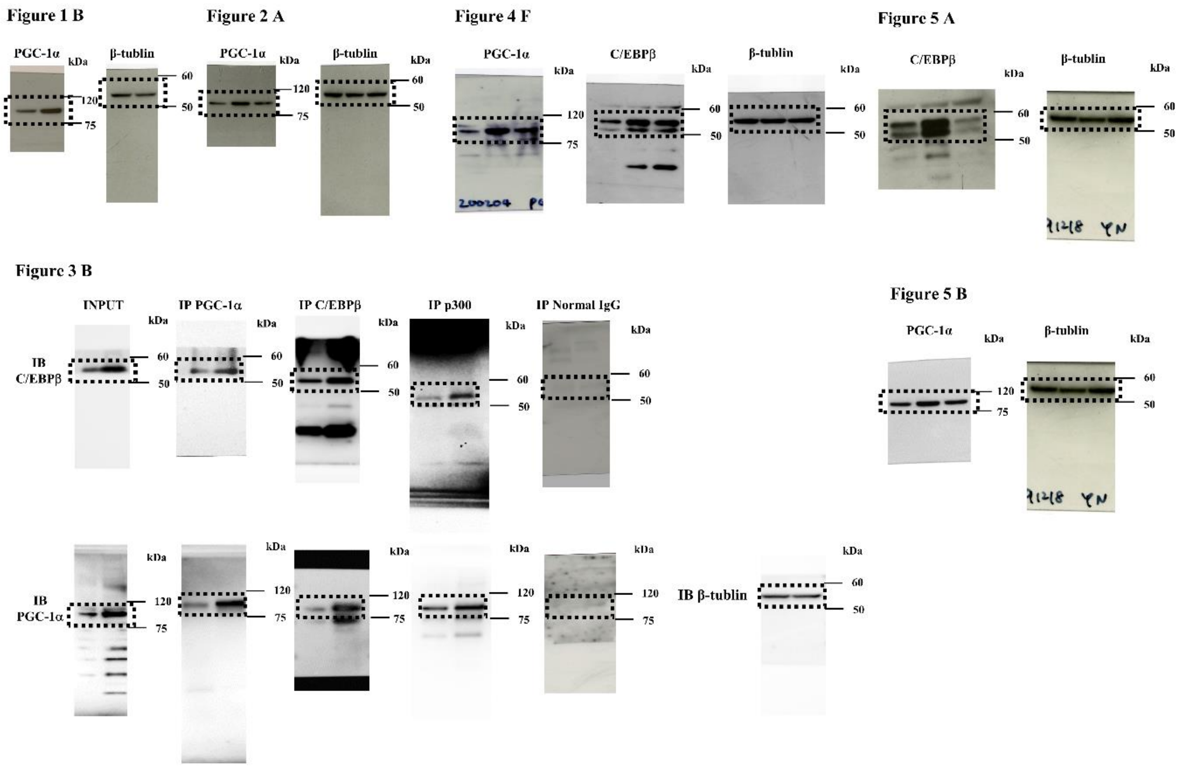

Figure S1. Uncropped images of the immunoblots of each figure. The cropped areas in each figure are indicated by dotted lines.
